# Influence of Dental Stages on Orofacial Muscle Strength and Oral Motor Behavior in Children

**DOI:** 10.1016/j.identj.2025.103870

**Published:** 2025-08-30

**Authors:** Linda Munirji, Abhishek Kumar, Ayumi Suzuki, Hanan Omairi, Joannis Grigoriadis, Anastasios Grigoriadis

**Affiliations:** a Karolinska Institutet, Department of Dental Medicine, Stockholm, Sweden; b Asahi University School of Dentistry, Department of Pediatric Dentistry, Mizuho, Gifu, Japan

**Keywords:** Mastication, Swallowing, Bite force, Tongue pressure, Development, TOMASS

## Abstract

**Aim:**

To study the effect of dental stages on orofacial muscle strength and masticatory and swallowing function in healthy children.

**Methods:**

A total of 120 children were recruited and divided into 6 groups according to Hellman's criteria for dental stages: primary, early mixed (IIC), early mixed (IIIA), late mixed, early permanent and late permanent. Each group underwent a series of tests to measure their orofacial muscle strength and masticatory and swallowing function. The data was analysed using one-way ANOVA followed by Tukey’s *post hoc* tests to adjust for multiple comparisons between successive dental stages.

**Results:**

The results of the study showed a gradual increase in orofacial muscle strength and improved masticatory and swallowing function with advancing dental stage. Specifically, significant differences between successive dental stages were shown in the maximum voluntary bite force between dental stages primary and early mixed (IIC), and between the early and late permanent, and in the tongue pressure between the primary and early mixed (IIC), and between the early mixed (IIIA) and late mixed. Additionally, in the test of masticating and swallowing solids, there were significant differences between dental stages primary and early mixed (IIC) in the number of chewing cycles, and between early (IIIA) and late mixed in the total eating duration.

**Conclusion:**

Overall, there was a gradual improvement in orofacial muscle strength with more developed dental stages in healthy children. The results of the study imply that the advancement of dental stages plays an important role in the development of orofacial muscle strength and improved masticatory and swallowing functions.

**Clinical Relevance:**

These findings suggest potential implications for monitoring the development of oral motor behaviours in growing children across different dental stages. The comprehensive battery of objective clinical tests applied in this study provides a valuable clinical framework for evaluating oral function.

## Introduction

The masticatory system is involved in different important functions such as chewing, swallowing, digestion, respiration and speech.^1^^,^^2^ The orofacial area that includes the masticatory system changes significantly during growth and development. Most notably, the dentition is transformed from primary to permanent. The intensive remodelling of craniofacial form and function occurs simultaneously with the long transition from the primary to the permanent dentition.^3^ The skeletal mass and shape, as well as the muscular mass, are profoundly altered during this transition, presenting a considerable challenge to the nervous system. Identifying developmental milestones and establishing normality indicators for oral functions in growing children is therefore essential.^3^^,^^4^

Sensorimotor integration is crucial for the precise control and coordination of oral functions such as biting, chewing, swallowing and speaking.^5^ This process involves continuous feedback between sensory input and motor output, allowing for smooth and efficient oral motor tasks.^6^^,^^7^ Children with primary dentition in particular exhibit immature motor control, characterised by a higher and more inconsistent force regulation during oral motor tasks.^3^^,^^8^^,^^9^ Nevertheless, as children grow through the late mixed dentition stage and into the early permanent dentition stage, their oral motor control undergoes significant changes and becomes more adult-like.^9^ This progression reflects the complex interplay of neurological and anatomical factors, highlighting the remarkable adaptability of the human oral sensorimotor system throughout childhood and adolescence.

Studies have shown that biting forces,^10^ tongue and lip muscle strength, and masticatory and swallowing performance tests are useful indicators of oral functions such as chewing and swallowing. It has been reported that maximum voluntary bite force (MVBF) and physiological parameters such as electromyography and kinematics of jaw muscles and chewing efficiency gradually change during development and are affected by the dental eruption stages.^3^^,^^11^ It was specifically shown that children with primary dentition had shorter lateral jaw movement and higher muscle activity at the end of the chewing sequence, in comparison with adults. Also, it was shown that children with primary dentition decreased their adaptation of jaw muscle activity to food hardness.^11^ Previous studies have assessed oral function or oral motor behaviour in children using one or more clinical tests, but to the best of our knowledge, no study has conducted a comprehensive oral function assessment comprising a battery of objective clinical tests in children at different dental developmental stages. Therefore, this study aimed to investigate the effect of different dental stages on the development of orofacial muscle strength and masticatory and swallowing functions in healthy children as they transition from primary to mixed and permanent dentition. The null hypothesis was that there was no significant effect of dental stages on the development of orofacial muscle strength and masticatory and swallowing function.

## Materials and methods

The study was conducted in accordance with the Declaration of Helsinki and approved by the Swedish Ethical Review Authority (Dnr 2018/726-31/2). Upon receiving verbal and written information about the study, participants—or their parents if they were younger than 15 years old—provided written consent to participate in the study. In addition, they were informed that they could discontinue the study at any time if they wanted to do so.

### Study participants

The study was carried out at the laboratory of oral rehabilitation in collaboration with the pedodontics dental clinic, Department of Dental Medicine, Karolinska Institutet, Stockholm, Sweden. G*Power was used to determine the required sample size for the current study based on earlier studies.^12^^,^^13^ With a significance level of α = 0.05, a power of 90% (1 – β = 0.90) and an effect size of 0.4, the analysis indicated that a total sample size of approximately 114 participants was needed. Therefore, 120 participants were recruited and divided into 6 groups with 20 participants each based on Hellman’s developmental dental stages: a classification system of dentition stages in children. A more detailed classification of the stages is presented in Table 1. The participants included were in good general and oral health, with no active caries or periodontitis. They exhibited no gross malocclusions and had not undergone any orthodontic or prosthodontic treatment. Furthermore, children were excluded from the study if they or their parents/guardians reported any history of serious systemic diseases, neurological disorders, temporomandibular disorders or orofacial pain.

**Table 1:** Demographic data of the participants and characteristics of Hellman’s dental developmental stages.

Hellman’s dental developmental stages		Participants (*n*)	Age (mean ± SD)	Age range (years)
Primary *Complete primary dentition*	IIA	20 (11 girls)	4.4 ± 0.9	3-6
Early mixed *First permanent molars eruptive phase*	IIC	20 (8 girls)	6.3 ± 0.8	5-8
Early mixed *First permanent molars fully erupted*	IIIA	20 (9 girls)	8.1 ± 1.1	6-10
Late mixed *Exchange of lateral teeth*	IIIB	20 (10 girls)	10.7 ± 1.4	8-13
Early permanent *Second permanent molar eruptive phase*	IIIC	20 (10 girls)	12.0 ± 1.2	9-14
Late permanent *Complete permanent dentition*	IVA	20 (10 girls)	15.2 ± 1.6	12-17

### Experimental protocol

The experimental session took about 30 minutes. The participants sat comfortably on an office chair, and children under 15 years of age had at least one parent present during the experimental session. The examiner carefully demonstrated all the tasks in the experimental session before starting. All the parameters used to measure orofacial muscle strength and oral motor behaviours have been standardised and extensively studied in previous studies.^14^, ^15^, ^16^, ^17^, ^18^

### Orofacial muscle strength

As a measure of orofacial muscle strength, the MVBF, tongue, cheek and lip pressure were measured individually. MVBF was measured using a custom-made, metal force transducer (Hottinger Brüel & Kjaer) adapted for intraoral use. For the comfort of the participant, a cotton layer was wrapped around the transducer and encased in a plastic bag. The transducer was positioned between the upper and lower first permanent molars (or the last primary molar if the first permanent molar had not yet erupted) on the participant’s preferred chewing side. Participants were instructed to clench their teeth as forcefully as possible without causing discomfort, and the MVBF was recorded in Newtons (N).

The tongue, cheek, and lip pressures were measured with the Iowa Oral Performance Instrument (IOPI) (IOPI Medical LLC). This instrument measures pressure in kilopascals (kPa) using a balloon (approximate size 3 cm x 1.5 cm x 1 cm). For tongue pressure measurement, the balloon was positioned on the participant’s tongue by the examiner, and the participant was directed to press the balloon against the hard palate as forcefully as possible without causing discomfort. Cheek pressure was evaluated by positioning the balloon in the oral vestibule near the first permanent molar on the participant’s preferred chewing side. Participants were instructed to press their cheeks against their teeth as forcefully as possible without causing discomfort. Similarly, to measure lip pressure, the examiner positioned the balloon between the participant’s upper and lower lips and instructed them to press their lips on the balloon as forcefully as possible without causing discomfort. Each of the orofacial muscle strength measurements was performed 3 times, and the average value was computed.

### Oral motor behaviours

A comprehensive masticatory function assessment was performed using masticatory efficiency, food comminution and a mixing ability test. Accordingly, the chewing efficiency test (CET) was performed using a standardised, hard viscoelastic test food, using a standardised recipe containing a mix of gelatine, water, sugar and glucose. For more details, please see previous studies.^11^^,^^16^ During the test, the participants were asked to consume the standardised test food by chewing and swallowing it. After they had swallowed the test food, the participants were asked to say their name loudly to indicate it was completely swallowed. The examiner silently counted the number of chewing cycles and the time taken to completely chew and swallow the test food. This test was video recorded so that the examiner could recount the time and chewing cycles later. The video was immediately deleted when the examiner had satisfactorily confirmed the number of chewing cycles and the time.

The same test food was also used to test the comminution ability in the food comminution test (FCT). During the test, the participants were asked to normally consume one piece of test food while the examiner silently counted the number of chewing cycles. The participants were then abruptly stopped and instructed to expectorate the pieces into a white petri dish after they had chewed the test food ten times. Water (30 ml) was added to the petri dish containing the test food, and if any pieces had adhered together, they were gently separated without causing breakage. A photographic image of the petri dish containing the expectorated test food was captured according to standardised conditions using a mobile phone (Samsung Galaxy A8™). The number of expectorated pieces of the test food was counted, with a higher count indicative of superior masticatory performance.

Additionally, the mixing ability was assessed with a two-colour chewing gum mixing ability test. Participants chewed on a two-coloured chewing gum (Vivident Fruitswing® “Karpuz/Asai Üzümü”; gum3) using their preferred chewing side.^17^ The examiner silently counted the chewing cycles and stopped the participant after 20 chewing cycles. The participants were then asked to expectorate the whole gum into a translucent resealable bag (6 × 9 cm), which was flattened to a wafer of one millimetre thick using two glass plates.^16^ An image of each side of the wafer was taken according to standardised circumstances using a phone (Samsung Galaxy A8™). The variance of hue (VOH) was calculated using the analysis software ViewGum (dHAL software). The lower the VOH, the superior the masticatory performance.

To assess swallowing function, the participants performed the test of masticating and swallowing solids in children (TOMASS-C).^18^ It is a validated test used for measuring food ingestion and swallowing efficiency of solid food.^19^, ^20^, ^21^ The participants were instructed to eat a cracker (Göteborg Saltiner^TM^) as quickly as comfortably possible and thereafter state their name out loud when the cracker was completely swallowed. The assessment captured four measurements: the number of discrete bites, chewing cycles, swallows, and the total duration to finish the cracker. This test was repeated twice, and the mean value for each measurement was calculated. Additionally, the test was video recorded to allow the examiner to recount and confirm the measurements. The video was immediately deleted after the recount.

### Statistical analysis

The statistical analysis was conducted using the statistical software program SPSS (IBM SPSS Statistics for Windows, Version 29). The Shapiro–Wilk test was used to check the assumption of normality. If the data were not normally distributed, a logarithmic transformation was applied. Subsequently, a one-way analysis of variance (ANOVA) was performed to analyse overall differences between dental stages, *post hoc* analyses were performed with Tukey’s Honestly Significant Difference test, and the differences between 2 successive dental stages during multiple corrections were presented. A *P* < .05 was considered statistically significant.

## Results

One hundred and twenty participants successfully participated in the study, divided into groups of 20 participants in each dental stage according to Hellman’s developmental stages. Demographic data for the participants in each dental stage are presented in Table 1. The results of ANOVA analysis for all parameters are presented, specifically highlighting the significant differences between successive dental stages.

### Orofacial muscle strength

The results from the ANOVA and the *post hoc* analysis for all variables measuring orofacial muscle strength, including MVBF and tongue, cheek and lip pressures, across different dental stages are shown in Table 2. The ANOVA showed significant main effects of dental stages on MVBF (*P* < .001), tongue pressure (*P* < .001) and cheek pressure (*P* < .001). However, there was no significant main effect of dental stages on lip pressure (*P* = .115) (Figure 1). *Post hoc* analysis comparing successive dental stages showed that MVBF was significantly higher in the early mixed (IIC) dental stage compared with the primary (*P* = .022) and the late permanent dental stage compared with the early permanent (*P* = .028) (Figure 1A). Additionally, tongue pressure was significantly higher in the early mixed (IIC) dental stage compared with the primary stage (*P* = .045) and in the late mixed dental stage compared with the early mixed (IIIA) stage (*P* < .001) (Figure 1B).

**Table 2:** Results of the ANOVA and post hoc analysis with Tukey’s Honestly Significant Difference test of the orofacial muscle strength and oral motor behaviour measurements.

Measurements		ANOVA	*Post hoc* analysis with Tukey’s Honestly Significant Difference test (*P*)				
		*P*	Primary (IIA) versus Early mixed (IIC)	Early mixed (IIC) versus Early mixed (IIIA)	Early mixed (IIIA) versus late mixed (IIIB)	Late mixed (IIIB) versus early permanent (IIIC)	Early permanent (IIIC) versus Late permanent (IVA)
Orofacial muscle strength	MVBF (N)	<.001*	0.022⁎⁎	0.495	0.892	0.167	0.028⁎⁎
	Tongue pressure (kPa)	<.001*	0.045⁎⁎	0.999	<0.001⁎⁎	0.407	0.325
	Lip pressure (kPa)	.115#	–	–	–	–	–
	Cheek pressure (kPa)	<.001*	0.975	1.000	0.833	0.807	0.997
Oral motor behaviours	CET – Chewing cycles(n)	<.001*	0.196	0.786	1.000	0.999	0.624
	CET – Time (sec)	<.001*	0.244	0.967	0.967	1.000	0.939
	FCT (n)	<.001*	0.938	0.184	0.989	0.998	0.376
	Mixing ability (VOH)	<.001*	0.980	0.353	1.000	0.961	0.125

⁎ Asterisks indicate significant main effects.

⁎⁎ Asterisks indicate significant differences between successive dental stages.

# No significant main effects.

**Fig. 1:**
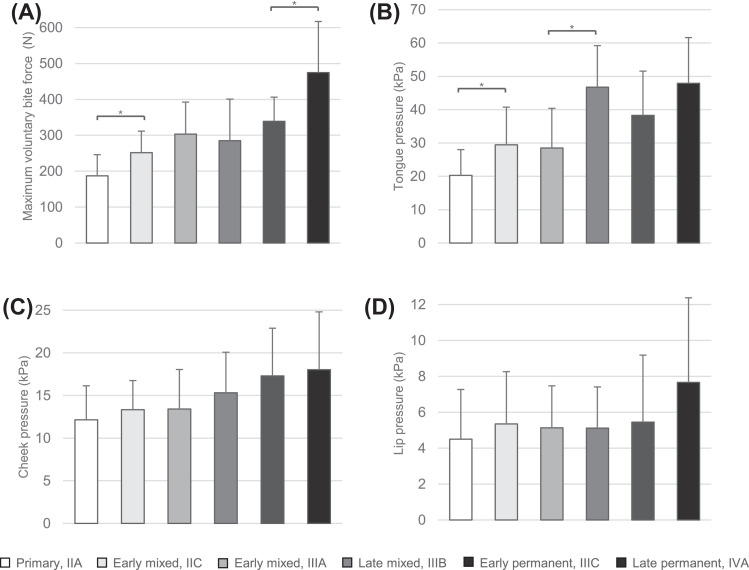
Bar graphs showing the mean values and standard deviations of the variables measuring orofacial muscle strength: (A) maximum voluntary bite force, (B) tongue pressure, (C) cheek pressure, and (D) lip pressure for each dental stage, specifically (1) primary, (2) early mixed IIC, (3) early mixed IIIA, (4) late mixed, (5) early permanent, and (6) late permanent. Asterisks indicate significant differences.

### Oral motor behaviours

**Fig. 2:**
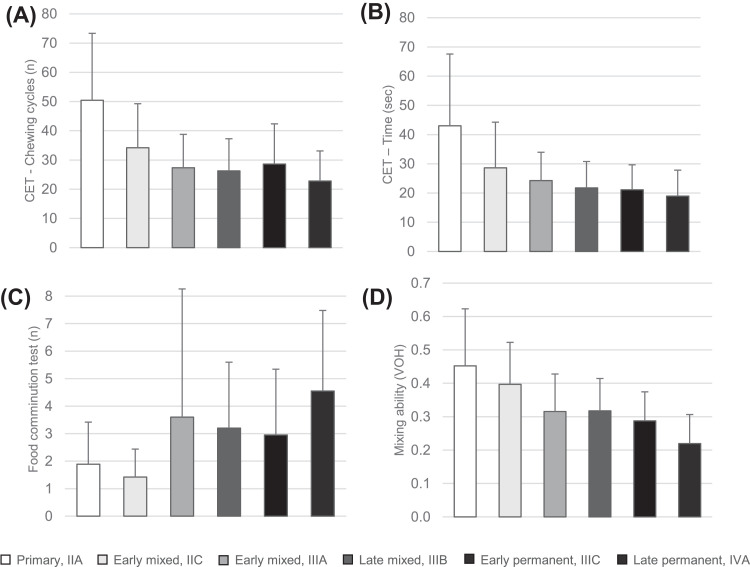
Bar graphs showing the mean values and standard deviations of the variables measuring oral motor behaviours: (A) number of pieces in the food comminution test, (B) chewing cycles in the chewing efficiency test (CET), (C) total duration of the CET, and (D) variance of hue in the gum mixing ability test for each dental stage, specifically (1) primary, (2) early mixed IIC, (3) early mixed IIIA, (4) late mixed, (5) early permanent, and (6) late permanent.

The results from the ANOVA and the *post hoc* analysis for all variables assessing masticatory function, including CET, FCT and mixing ability with chewing gum, across dental stages are presented in Table 2. There were significant main effects of ANOVA across dental stages in CET (*P* < .001), FCT (*P* < .001) and the mixing ability test with chewing gum (*P* < .001) (Figure 2). However, *post hoc* analysis showed no significant differences between successive dental stages in any of the tests.

**Fig. 3:**
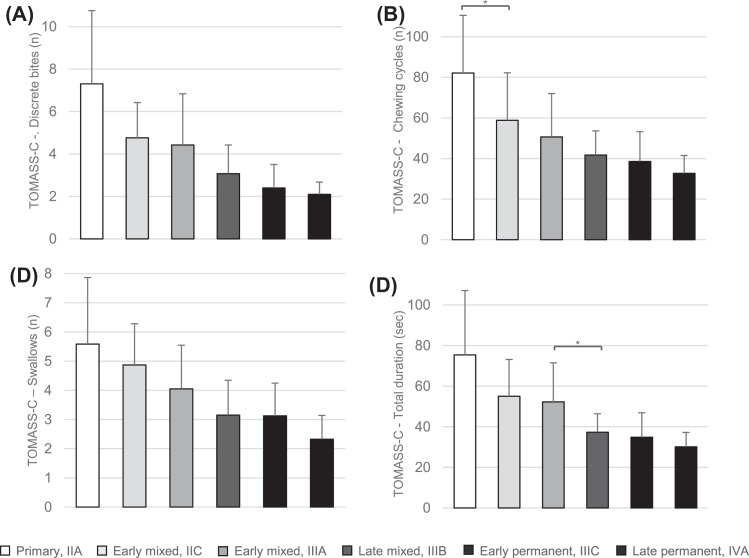
Bar graphs showing the mean values and standard deviations of the measured variables in TOMASS-C: (A) number of discrete bites, (B) number of chewing cycles, (C) number of swallows, and (D) total duration until completion of eating the cracker for each dental stage, specifically (1) primary, (2) early mixed IIC, (3) early mixed IIIA, (4) late mixed, (5) early permanent, and (6) late permanent. Asterisks indicate significant differences.

The results from the ANOVA and the *post hoc* analysis for all variables in TOMASS-C, including the number of discrete bites, chewing cycles, swallows and total duration for completely consuming the cracker, are presented in Table 3. ANOVA revealed significant main effects between different dental stages in the TOMASS-C regarding all four variables (*P* < .001). *Post hoc* analysis revealed a significant difference between two successive dental stages in the number of chewing cycles, between dental stages primary and early mixed (IIC) (*P* = .033) (Figure 3B) and in the total duration for completing the cracker between dental stages early mixed (IIIA) and late mixed (*P* = .029) (Figure 3D). However, no other significant differences were found between successive dental stages in the other variables.

**Table 3:** Results of the ANOVA and *post hoc* analysis with Tukey's Honestly Significant Difference test for the TOMASS-C.

TOMASS-C measurements	ANOVA	*Post hoc* analysis with Tukey's Honestly Significant Difference test (*P*)				
	*P*	Primary (IIA) versus Early mixed (IIC)	Early mixed (IIC) versus Early mixed (IIIA)	Early mixed (IIIA) versus late mixed (IIIB)	Late mixed (IIIB) versus early permanent (IIIC)	Early permanent (IIIC) versus Late permanent (IVA)
Discrete bites (*n*)	<.001*	0.067	0.938	0.086	0.415	0.986
Chewing cycles (*n*)	<.001*	0.033⁎⁎	0.550	0.696	0.924	0.801
Swallows (*n*)	<.001*	0.956	0.557	0.199	1.000	0.160
Total duration (sec)	<.001*	0.075	0.983	0.029^⁎⁎^	0.951	0.811

⁎ Asterisks indicate significant main effects.

⁎⁎ Asterisks indicate significant differences between successive dental stages.

## Discussion

The study investigated the effect of different dental stages on the development of orofacial muscle strength and masticatory and swallowing function in healthy children. In general, the results showed a significant increase in orofacial muscle strength and a more efficient masticatory and swallowing function with a more developed dental stage, in accordance with previous studies.^22^, ^23^, ^24^, ^25^ Specifically, there was a significant increase in MVBF between the primary dental stage and the early mixed dental stage, as well as between the early permanent stage and the late permanent stage. Similarly, tongue pressure was significantly higher in the early mixed stage compared to the same in the primary stage, and in the late mixed stage compared to the early mixed stage. Additionally, the number of chewing cycles in TOMASS-C significantly decreased in the early mixed dental stage compared to the same in the primary stage, and the total duration of chewing and swallowing the cracker significantly decreased in the late mixed dental stage in comparison to the same in the early mixed dental stage. These findings suggest that dental development can influence orofacial muscle strength and masticatory and swallowing functions. Therefore, the null hypothesis was rejected.

A comprehensive evaluation of orofacial muscle strength and oral motor behaviours using a battery of easy-to-use and objective clinical assessments is essential for accurately determining how dental developmental stages influence oral functions. Isolated clinical tests may provide valuable insights into specific aspects of oral function, such as masticatory performance, tongue strength or swallowing ability.^2^^,^^26^ However, a holistic approach using multiple complementary assessments allows for a more nuanced understanding of the complex interactions between dentition, orofacial muscles and neuromotor control. This may be particularly important in children, in whom oral function evolves dynamically with growth and dental development.^9^^,^^11^ Research shows that muscle strength in children varies significantly under the influence of factors such as growth spurts, muscle mass, biological maturity and neuromuscular adaptations.^27^^,^^28^ Older children usually have more developed muscles and advanced neuromuscular coordination.^27^ However, the development of oral functions may occur at different rates^29^^,^^30^ and may be influenced by the primary dentition transitioning to the permanent. Further, age and individual variations in tooth eruption patterns can affect children in different dental stages despite being in the same age group. This transition period is essential for the development of oral functions such as chewing, swallowing and speaking.

Clinical studies often have limitations, and the ones in this study need to be acknowledged. One such limitation was the challenges around measuring lip pressure, especially for younger children. It was observed that at times children found it difficult to comprehend the instructions for pursing their lips to accommodate the balloon transducer of the IOPI between their lips. Also, it was observed that some children would bite the device instead of pressing their lips together. However, the examiner closely monitored the task and re-explained it when necessary, and all but 1 participant completed the task correctly. The test food used in the current study was custom-prepared using a standardised recipe that has been used in several previous studies.^11^^,^^16^^,^^31^, ^32^, ^33^ However, with gelatine being one of the ingredients, some participants abstained from eating it. Therefore, future studies should consider alternative ingredients and develop gelatine-free test food. At the same time, only 3.3% of the participants abstained from eating the test food, and we believe this small percentage is unlikely to have impacted the study’s outcome to a significant degree. It can also be argued that the size of the test food was the same across all age groups, and that, having comparatively smaller oral cavities, younger children may have demonstrated signs of poor chewing performance because of this^11^; however, the dimensions of the test food (20 × 10 mm) were comparable to the common confectioneries consumed by all children regardless of age, and thus unlikely to have been a factor affecting chewing performance.

In agreement with earlier studies,^25^^,^^34^ the MVBF in our study increased significantly between different dental stages, which could be attributed to the increased number and area of occlusal contacts, as well as the overall development and maturation of the masticatory muscles. Castelo et al. suggested in their longitudinal study^25^ that facial morphology and dental stage development have a more significant impact on MVBF than age or body mass index. The development of the masseter muscle and changes in facial dimensions, particularly in the vertical dimension, are more closely linked to increases in MVBF as children transition from primary to mixed dental stages.^25^ Similarly, our data show a significant increase in MVBF between primary and early mixed dental stages, where the first key transition occurs. By the late permanent dental stage, the participants had a 2.5-fold higher MVBF than in the primary dental stage. This aligns with earlier findings, showing that children between ages 7 and 12 had significantly lower MVBF in comparison to those aged 13 and older, which also remained stable during adulthood, until around 60 years of age.^35^ Further, according to previous research, a mature, adult-like chewing pattern develops around age 12.^11^^,^^36^ Our data corroborate these findings, showing a significant increase in MVBF in the late permanent dental stage compared with the early permanent. This indicates that once children reach their late permanent dental stage, the MVBF is stable during adulthood.

Tongue pressure is a common and reliable measurement to evaluate oral function. The tongue plays an important role in forming a food bolus after chewing and leading it towards the pharynx for swallowing.^37^ In normal mature swallowing, the tongue makes precise, coordinated movements to ensure food is adequately processed and transported. In infants, the tongue is positioned more forward, which leads to a prolonged late transport phase and faster tongue movements in the early final phase of swallowing. As children grow, the tongue retracts and muscle coordination improves, transitioning to a more mature swallowing pattern. This shift is typically complete between the ages of 2 and 4 years.^38^ Problems with tongue strength in children, similar to adults, can directly affect the oral phase of swallowing, potentially causing dysphagia.^39^^,^^40^ A study measuring tongue pressure showed that there is a rapid increase in tongue pressure from age 3 to age 8, with a continuing increase at a slower rate with increasing age during childhood until late adolescence and young adulthood, at which point it reaches its peak.^39^ Our study shows similar results, with a rapid and significant increase between primary (mean age 4.4) and early mixed (mean age 6.3) dental stages as well as between early mixed (mean age 8.1) and late mixed (mean age 10.7) dental stages, with the results in late mixed being almost as high as the most developed dental stage, the late permanent (mean age 15.2) (Figure 1B). Our data also show that tongue pressure more than doubles in the late mixed dentition in comparison to the primary dentition. The earlier studies demonstrate a 2-3 times lower tongue pressure in 3-year-olds in comparison to 10-11-year-olds,^39^^,^^41^ aligning with the average age of the participants in the late mixed dentition in our study.

The lip closing function is complex, requiring various movements, and could affect several functions, including chewing, swallowing and breathing.^42^^,^^43^ In our study, lip pressure was the only parameter that did not show significant main effects between dental stages. This aligns with the findings of Lambrechts et al., who also observed no significant age-related differences in lip pressure in children.^44^ Cheek pressure, on the other hand, showed significant main effects between different dental stages, but not between successive dental stages.

The viscoelastic test food used in the FCT and CET is a good tool for evaluating masticatory function and efficiency. It provides effective mechanical challenges and enhances high sensorimotor control during food breakdown.^16^ The mixing ability of chewing gum has also been shown to be a valuable tool for evaluating chewing performance.^17^^,^^45^ In the current study, there was a significant increase in masticatory performance with more advanced dental stages, reflected in less time and fewer chewing cycles in CET, a higher number of expectorated pieces in the FCT, and better mixing ability with chewing gum, aligning with the earlier studies.^46^^,^^47^ All children, regardless of dental stage, were given the same-size piece of viscoelastic test food, which was similar in size and taste to a standard piece of candy. Even though some of the children tended to swallow the food without optimum chewing, they still participated enthusiastically and were able to eat the whole test food. According to Almotairy et al., children do not divide their test food into smaller pieces, as adults do, before swallowing, which leads them to swallow bigger pieces of food, calling it ‘quite a mouthful’.^11^ Almotairy et al. also suggested that the more developed the dental stage, the less time is needed for splitting the food,^9^ correlating with our findings that children with a more advanced dental stage achieve a higher number of pieces. As previously mentioned, a more mature swallowing pattern often develops between the ages of 2 and 4,^38^ and an adult chewing pattern develops around the age of 12,^36^ which may be the reason why younger children are not able to split the food the way older children can, although they are able to swallow it without issues.

To quantify solid food ingestion, TOMASS for children^18^, ^19^, ^20^ was conducted, and the results show significant effects across different dental stages. However, a *post hoc* analysis indicated a significant difference between successive dental stages only in the number of chewing cycles between primary and early mixed dentition and in the total duration of completing the cracker between the early mixed and late mixed dental stages. Frank et al. reported normative values for the TOMASS-C across various age groups, showing a gradual increase in performance with age.^18^ Our results show a similar increase with more developed dental stages (Figure 3).

## Conclusion

The results of the study showed a significant increase in orofacial muscle strength and more efficient masticatory and swallowing functions with advancing dental stages. MVBF and tongue pressure significantly increased from primary to early mixed dental stages, and the number of chewing cycles in TOMASS-C significantly decreased as the dental stages advanced. Additionally, MVBF also increased from early to late permanent stages, while from early to late mixed dental stages, tongue pressure increased and the total time duration for completing the cracker in TOMASS-C decreased. These findings indicate that orofacial muscle strength and oral motor behaviours associated with masticatory and swallowing ability in children are influenced by dental developmental stages. By conducting multiple objective clinical tests, this study provided a comprehensive assessment of oral function among children in different dental developmental stages. These results also suggest potential applications in monitoring the development of oral functions in growing children across different dental stages.

## Author contributions

Conceptualization: A. Grigoriadis, J. Grigoriadis, Kumar

Data collection: Munirji, Omairi, Suzuki

Data curation: A. Grigoriadis, Kumar

Data processing: Munirji, Suzuki

Formal analysis: A. Grigoriadis, J. Grigoriadis, Kumar, Munirji

Project administration: A. Grigoriadis

Supervision: A. Grigoriadis, J. Grigoriadis, Kumar

Writing—original draft: Munirji

Writing—review and editing: All authors

## Funding

Partial financial support was received from SOF (Styrgruppen för Odontologisk Forskning).

## Conflict of interests

None declared.
